# Synergistic and concentration-dependent toxicity of multiple heavy metals compared with single heavy metals in *Conocarpus lancifolius*

**DOI:** 10.1007/s11356-020-12271-0

**Published:** 2021-01-14

**Authors:** Amina Redha, Redha Al-Hasan, Mohammad Afzal

**Affiliations:** 1 Department of Biological Studies, Faculty of Science, Kuwait University, Kuwait City, Kuwait; 2 Gainesville, USA

**Keywords:** *Conocarpus lancifolius*, Heavy metal, Bioaccumulation factor, Enrichment factor, Translocation factor, Oxidative stress

## Abstract

While heavy metals (HMs) naturally occur in soil, anthropogenic activities can increase the level of these toxic elements. *Conocarpus lancifolius* Engl. (Combretaceae) was investigated as a potential phytoremediator of soils contaminated with HM containing crude oil. This study assessed the potential of *C. lancifolius* (CL), a locally available plant species in Kuwait, for resolving local issues of the HM-contaminated soils. The absorption, accumulation, and distribution of three toxic HMs (Cd, Ni, and Pb) and essential metals (Fe, Mg, and metalloid Se) were examined, and their role in plant toxicity and tolerance was evaluated. *Conocarpus lancifolius* plants were exposed to two different concentrations of single and mixed HMs for 30 days. The accumulation of HMs was determined in the roots, leaves, stems, and the soil using ICP/MS. Biomass, soil pH, proline and protein content, and bioaccumulation, extraction, and translocation factors were measured. The bioaccumulation, extraction, and transcription factors were all >1, indicating CC is a hyperaccumulator of HM. The HM accumulation in CL was concentration-dependent and depended on whether the plants were exposed to individual or mixed HMs. The C.C leaves, stems, and roots showed a significant accumulation of antioxidant constituents, such as proline, protein, Fe, Mg, and Se. There was an insignificant increase in the soil pH, and a decrease in plant biomass and a significant increase in protein, and osmoprotective-proline as a result of the interaction of mixed heavy metals that are more toxic than single heavy metals. This study indicates that *C. lancifolius* is a good candidate for phytoremediation of multiple HM-contaminated soils. Further studies to establish the phyto-physiological effect of multiple heavy metals are warranted.

## Introduction

During the extraction and transport of crude oil, heavy metals (HMs) become an important component of the soil, and because HMs are nondegradable, they persist in the environment and they impose potential human health and ecological risks (Lian et al. 2019; Yan et al. 2020). In response to oxidative stress (OS), HMs cause a steady rate of the production of reactive oxygen species (ROS) causing extensive cellular damage to the live organisms (Gao et al. 2019; Hasanuzzman et al. 2020). The production of ROS reduces the antioxidant defenses, which negatively affects cytoplasmic enzymes and causes a serious impairment of cellular structure, ultimately leading to physicochemical changes in the plants (Shahid et al. 2014; Nanda and Agrawal 2016; Laxa et al. 2019). HMs can also damage the cell wall proteins and nuclear DNA (Nanda and Agrawal 2016), and interact with the central Mg^2+^ in chlorophyll, thereby disrupting photosynthetic activity. The damage can be aggravated by altering the morphology and growth dynamics of plants and the damage places reliance on the rate of accumulation of the HMs (Küpper et al. 2006). Plants have dissimilar HM-accumulation capacities, and species with higher amassing potentials, such as *Brassica juncea* (Brassicaceae) subsp. *Integrifolia*, Var. *strumata* and *B. napus*, have been used for the phytoremediation of HM-contaminated ecosystems (Mourato et al. 2015).

Phytoremediation of soil is cost-effective involving green chemistry, but its success depends on many factors, such as plant species, soil texture, and its pH (Farrag et al. 2011). In response to abiotic stresses, plant roots exude a diverse array of chemicals that may support unique rhizospheric microbial communities, essential for plant survival, and growth (Skowronska et al. 2020). Therefore, an adequate balance is established between rhizospheric bacteria, HMs, and root exudates, which determines the uptake of HMs and their translocation by the roots to the overground parts of the plant. The root hairs increase the root surface area and play a primary role in the plant-soil interactions that can regulate the hydrodynamics and absorption of HMs from the soil.

The most important parameters for the effective phytoremediation include rapid biomass production and effective translocation of pollutants into all parts of the plant (Rezvani and Zaefarian 2011). Mahajan and Kuashal (2018) have described four criteria for phytoremediation: (a) a rapid growth rate, (b) a high biomass, (c) a hairy deep-root system, and (d) a bioaccumulation factor (BAF) > 1.

*Conocarpus lancifolius* tree is native to riverine areas of Somalia, Djibouti, Yemen, horn of Africa, Arabian Peninsula, and South Asia. Before the 1991 Gulf war, this tree was not found in Kuwait desert. For greenery projects undertaken by the government of Kuwait, *C. lancifolius* was introduced into Kuwait from Djibouti. In the harsh environment of Kuwait desert, the plant has thrived well and now it is found along all main boulevards of the city, parks, and is used as a green fence around private houses. It is also trimmed into various attractive shapes that enhance its grace. *C. lancifolius* has a fast growth, long hairy roots, high availability, approval by the local population, and due to its large biomass that provides substantial quantities of shading material.

A major catastrophe occurred in land, marine, and coastal environments during the Gulf war in 1991, when 6–8 million barrels of crude oil were spilled into the marine environment of Kuwait waters. In addition, setting oil wells ablaze propelled massive amounts of soot and toxic gasses into the atmosphere, causing one of the biggest man-made ecological disasters in human history. The spilling of crude oil imposed deleterious effects on the ecosystem, due to the persistent toxicity of several HMs present in oil residues. However, after thirty years of the war, along the Wafra road (an oil production area), the soil marginally contains Cd at 0.027 mg kg^−1^ soil, Ni at 120.96 mg kg^−1^ soil, and Pb at 2.9 mg kg^−1^ soil, levels well below the contaminated soils (Kostecki and Behbehani 1995; Wuana and Okieimen 2011; Chinedu and Chukwuemeka 2018).

In 2009, we initiated a systematic study on *C. lancifolius* and discovered that the plant was resistant to drought conditions and high salinity, and it could endure high desert temperatures (Redha et al. 2011, 2019). These characteristics, fast growth rate, and its large biomass make this plant a good candidate for phytoremediation studies involving HM-contaminated soil. In polluted soils, metals are not present as single elements, but they are a mixture of metals affecting flora and fauna of the environment. Although the effect of heavy metals on *C. lancifolius* irrigated with wastewater has been reported (Rasheed et al. 2019), the objective of this investigation was to demonstrate the assimilation of multi-metals by this plant and upregulation of its antioxidant defenses. Since *C. lancifolius* meets all the benchmarks of a good phytoremediator, we decided to examine its incorporation of multi-metals from the oil-polluted soil of Kuwait.

## Materials and methods

### Plant materials and growth conditions

All chemicals used in this study were purchased from VWR International (Solsbury, UK). According to WRB classification, the Kuwait local soil is Regosol in the arid desert and Flavisol in some northern coastal areas.

Plants were obtained from the Public Authority for Agriculture and Fish Resources (PAAFR; a government organization in the State of Kuwait). The growth and maintenance conditions of the plants were followed as reported earlier (Redha et al. 2019). Local sandy soil and peat moss (3:1 v/v; 3.5 kg) were used for plant growth in plastic pots (9 × 6"). Seventy plants of uniform height, with 10–12 leaves and a single shoot, were maintained at 25°C, with a relative humidity of 45–55%, and a white light intensity of 150 mmol quanta m^−2^ s^−1^ for 30 d. Plants were divided into 11 groups (a–k) with six plants in each group. Group-a served as the control without HM solution treatment. Plants in groups b–k were irrigated with 50 mL of 25 or 50 μmol L^−1^ of the single HM solutions (Cadmium (II) nitrate tetrahydrate, Nickle (II) nitrate hexahydrate, and Lead (II) nitrate salts), or 50 mL of the 25 μmol L^−1^ mixed HM solutions on alternate days. Plant groups-b, d, and f were treated with 25 μmol L^−1^ of Cd, Pb, and Ni, respectively; plant groups-c, e, and g were treated with 50 μmol L^−1^ of Cd, Pb, and Ni, respectively, and plant groups h–k were treated with 25 μmol L^−1^ of mixed HMs (Cd-Pb, Cd-Ni, Ni-Pb, or Cd-Ni-Pb, respectively). Plants did not survive when irrigated with mixed HMs at 50 μmol L^−1^. Leaf and stem samples were collected on days 10, 20, and 30 of the experiment, and root samples were collected on day 30. The plant samples were thoroughly washed with distilled water to remove any soil particles, and oven-dried at 70°C for 24 h (Thermo Fisher Scientific, Rochford, UK, Model ELED 3625A-1) to a constant weight. The dried plant tissues were powdered and weighed. The HMs (Cd, Pb, and Ni) and essential metals (Fe, Mg, and metalloid Se) in 1 g of powdered plant tissue were measured by inductively coupled plasma/mass spectrometry-mass spectrometry (ICP/MS, details under ICP/MS measurements).

### Microwave digestion of the plant material

Plant material samples were prepared according to Kisku et al. (2000), using both high temperature and pressure to ensure total HM extraction. Dry powdered plant material (1 g, root or leaf) was placed in the reaction vessel and a mixture of concentrated nitric acid (65%) and concentrated perchloric acid (4:1 v/v), was added to the reaction vessel (EPA method 3052). The reaction vessel was sealed and heated in a microwave oven (Gallenkamp 300 plus electric oven, Loughborough, UK). The digestion was completed at (180–1000°C) for 20 min, with ventilation at 20 min. The reaction contents were transferred into a 25 mL polypropylene flask, and the digestion vessel was thoroughly washed with milli-Q water, and this was mixed with the sample digestion reaction contents. Reagent blanks were prepared using the same methods as for the samples. A high-purity concentrated nitric acid (HNO_3_; 69–70%, extra-quality, Fisher Scientific, and certified 30% hydrogen peroxide (Analytical Reagent Grade, BDH Ltd, Poole, UK) were used. Double distilled deionized water (Milli-Q Millipore 18.2 MΩ cm^−1^ resistivity) was used for all dilutions. Plant samples were prepared in triplicate (18 samples from each group), and the respective means and standard errors of the means (± SEMs) were used in analyses.

### ICP-MS measurement of Cd, Pb, Ni, Fe, Mg, and Se in leaves, roots, and soil samples

Dried leaf, stem, root, and soil samples (1 g each) were individually used to determine the HM contents, and results were expressed as μg g^−1^. A Perkin Elmer inductively coupled plasma-mass spectrometer (ICP-MS-Perkin Elmer Optima 7300DV, San Francisco, CA, USA) was used to detect HMs. We report the accumulation of HMs in the treatment groups relative to the HM levels in the control.

The ICP-MS system was calibrated with certified reference materials (CRM), Rhodium (Rh), and Rhenium (Re) nitrate salts ( Sigma-Millipore-Supelco, USA). The reagent blank solution contained 1% concentrated HNO_3_. Mixed standard solutions (reagent blanks) contained six elements (Mg, Se, Fe, Ni, Pb, and Cd). The background interferences from the plasma gasses, i.e., air entrainment and solvent, were corrected by subtraction of reagent blank signals. To monitor the drift of the instrument, the CRM element Yttrium, Y(NO_3_)_3_ (Sigma-Millipore-Supelco, USA), was used as an internal standard.

The instrumental operating parameters were as follows: plasma gas flow, 15 L min^−1^; nebulizer gas flow, 0.97 L min^−1^; lens voltage, variable; dual detector; cross flow nebulizer; flow rate of sample, 1 mL min^−1^; and spectra scanning, peak hopping. The instrument calibration was done using standard solutions, and a calibration curve was created. Quality control checks, internal calibration verifications (standards from different vendors), internal calibration blanks, continuing calibration verifications, and blank samples were run before and after the analyses of experimental samples. The HM content (mg kg^−1^) was calculated using the following equation:

$$ \mathrm{HM}\ \mathrm{content}=\mathrm{Instrument}\ \mathrm{reading}\times 50\times 10 $$

where 50 is the final sample volume, and 10 is the dilution factor.

### Measurement of bioaccumulation, enrichment, and translocation factors

The BAFs, EFs, and TFs were used as markers to appraise the capacity of plants to eliminate HMs from the soil and plant-soil interactions (Kisku et al. 2000; Kumar et al. 2018; Sampanpanish and Nanthavong 2019). These factors were calculated according to Rezvani and Zaefarian, (2011), using equations 1–3. All metrics were measured on the 30^th^ day of the experiments. The source of the HMs in the control plants was the contaminated soil in which the plants were grown.

1 $$ \mathrm{BAF}=\frac{\mathrm{Metal}\ \mathrm{content}\ \mathrm{in}\ \mathrm{contaminated}\ \mathrm{leaf}/\mathrm{stem}}{\mathrm{Metal}\ \mathrm{content}\ \mathrm{in}\ \mathrm{contaminated}\ \mathrm{soil}} $$

2 $$ \mathrm{EF}=\frac{\mathrm{Metal}\ \mathrm{content}\ \mathrm{in}\ \mathrm{contaminated}\ \mathrm{leaf}/\mathrm{stem}}{\mathrm{Metal}\ \mathrm{content}\ \mathrm{in}\ \mathrm{control}\ \mathrm{leaf}} $$

3 $$ \mathrm{TF}=\frac{\mathrm{Metal}\ \mathrm{content}\ \mathrm{in}\ \mathrm{contaminated}\ \mathrm{leaf}/\mathrm{stem}\ }{\ \mathrm{Metal}\ \mathrm{content}\ \mathrm{in}\ \mathrm{contaminated}\ \mathrm{roots}\ } $$

Accumulator and excluder plants accumulate metals at >1 mg kg^−1^ and <1 mg kg^−1^, respectively. The BAFs, EFs, and TFs for hyperaccumulators have been reported to be >1 (McGrath and Zhao 2003). Hyperaccumulators are known to accumulate 50–500 times more metals than ordinary plants (Chaney et al. 1997).

### Soil pH, biomass, proteins, and proline measurements

Soil samples (20 g) were collected on days 10, 20, and 30 from control pots (without exposure to HMs) and from pots exposed to HMs. Each soil sample was mixed with 40 mL of distilled/deionized water (50 mL) for 30 min, and the pH of the supernatant was measured with a calibrated pH meter (pH 4 and 7, Cole-Parmer Model 430; Eaton Socon, Saint Neots, UK). The pH was recorded in triplicate (i.e., from three soil samples) per pot, and the mean ± SEMs were calculated. Biomass was measured by drying six individual plants per treatment at 75°C for 48 h, recording their individual weights, and calculating the treatment means ± SEMs. The total proteins in the freeze-dried plant were measured according to Bradford (1976).

### Protein determination

A standard solution of bovine serum albumin solution (0.1 mg/mL) was prepared, and Bradford reagent (Sigma-Aldrich B6916, St. Louis, MO-63103, USA) was used for all protein assays. Absorbance was taken at 595 nm and a linear equation (*R*^2^ = 0.9991) was generated with different concentrations of the standard protein solution, and it was used to calculate protein in the plant extracts prepared as given below.

Fresh freeze-dried plant tissue (100 mg) was homogenized with a polytron homogenizer (PT10/35 GT, Kinenatica, Zaragoza, Spain) in PBS 1X (5 mL) and centrifuged (Bench top, Sorvall ST 8, Thermo Fisher Scientific, Rochford, UK) at 10,000*g* for 15 min. The supernatant was taken and the pellet was twice washed with deionized water and centrifuged. The combined supernatant was diluted with deionized water to make the volume to 100 mL, and its absorbance was used in the linear equation to calculate the protein concentration as μg/g plant material. This procedure was repeated in triplicate on three different samples of the same plant tissue and SEM was taken.

### Proline determination

Proline was measured using spectrophotometry as described in the literature (Carillo and Gibon 2011, Bergman and Loxley 1970). Briefly, proline standard solution (0.01-0.04 mM) was prepared in ethanol : water (70 : 30 v/v). The reaction mixture was prepared by mixing ninhydrin 1% (w/v) in acetic acid 60% (v/v) and ethanol 20% (v/v). The 400 μl of standard proline solution was mixed with 100 μl ninhydrin reaction mixture and heated to 95°C for 20 min. After cooling and spinning for 1 min at 2500 rpm, the absorbance was taken at 520 nm. A standard curve was obtained and the generated linear equation (*R*^2^ = 0.9993) was used to calculate proline in plant extracts.

Plant extract was prepared from 0.5 g fresh frozen plant tissue, and thawed and homogenized with a polytron homogenizer (PT10/35 GT, Kinematica, Zaragoza, Spain) in 5 mL ethanol : water (70 : 30 v/v) and centrifuged (PT10/35 GT, Kinematica, Zaragoza, Spain) at 10,000*g* for 15 min. The supernatant was taken and the pellet was twice re-extracted with 5 mL of the same solvent mixture. The combined supernatant was diluted to 100 mL with the same solvent mixture. The ninhydrin reaction mixture, prepared as above, (100 μl) was mixed with the plant extract (400 μl) and absorbance (520 nm) was used in the linear equation to calculate proline concentration. This procedure was repeated in triplicate on three different samples and SEM was taken.

### Statistical analysis

A one-way nonparametric ANOVA, followed by Newman-Keuls post hoc analysis, was used for data analysis. A two-tailed unpaired Student’s *t*-test was used to compare two groups. Each parameter was estimated three times and the mean ± SD values were recorded. The independent variables were the time of exposure and metal concentration. All statistical analyses were performed using the GraphPad Prism software (Version 8.4.3; San Diego, CA, USA), and *p* values < 0.05 were considered statistically significant. All tests were conducted on the data collected on the 30^th^ day of the experiment.

## Results

**Fig. 1:**
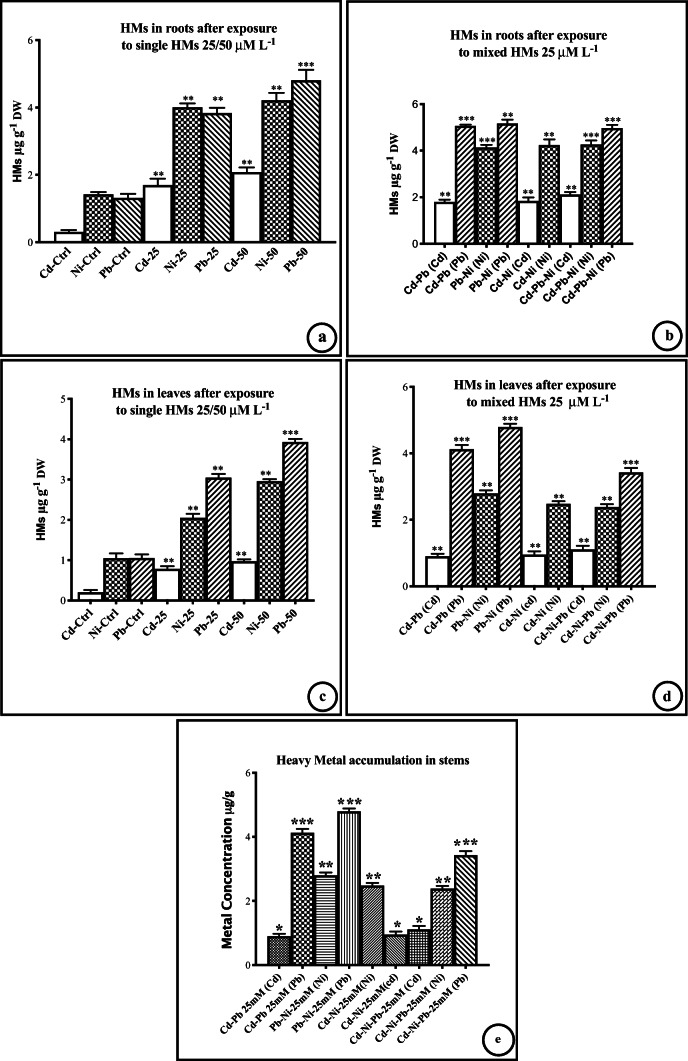
Uptake of single/mixed heavy metals in roots and leaves at two different concentrations: Uptake of heavy metals in roots **a**, **b**, leaves **c**, **d**, and stems **e** after *Conocarpus lancifolius* plants were exposed to single (25 or 50 μmol L^−1^) and mixed heavy metals (HMs) 25 μmol L^−1^. **d** The metal enclosed in brackets represents the metal that was measured in the mixture. Data were compared with the respective control data and a one-way ANOVA was used to assess differences between the means and *p* values. Bars and error bars represent means ± SEMs. Ctr, control group; the number after the metal symbol represents the concentration in μmol L^−1^; **p, significant differences (*p* < 0.01); ***p, highly significant differences (*p* < 0.001)

The treated *C. lancifolius* plants survived single metal treatment at both 25 and 50 μmol L^−1^ for 30 d. However, the plants did not survive after exposure to mixed HMs at 50 μmol L^−1^. Groups b, d, and f exposed to 25 μmol L^−1^, and groups c, e, and g exposed to 50 μmol L^−1^, differed significantly in their heavy metal accumulation (*p* < 0.0001, F values: Pb 3168, Ni 1640, Cd 275.6; ANOVA). The Newman-Keuls multiple comparison test showed a significant difference (ANOVA, *p* < 0.0001, F ratio = 275.6, df = 17) between the control plants (group-a) and the experimental groups-b, d, and f and groups-c, e, and g, in terms of HM accumulation. In all treatment groups exposed to HMs at 25 μmol L^−1^, the accumulation of HMs was significantly higher in the roots than in the leaves (*p* < 0.00001, ANOVA, df = 17). Compared with the control plants, plants exposed to single HMs at 50 μmol L^−1^ (groups-c, e, g) had a significantly higher HMs accumulation than those exposed to single HMs at 25 μmol L^−1^ (groups-b, d, f; ANOVA, *p* < 0.0001, Bartlett’s statistic, 16.64, df = 2), revealing the effect of HM concentration on HM accumulation both in the leaves and roots. Plants exposed to the mixed HMs at 25 μmol L^−1^ (groups-j–k; Fig. 1b, d) showed an increase in HM accumulation that was similar to plants exposed to single HMs at 50 μmol L^−1^ (groups-c, e, g; Fig. 1a, c). The accumulation of the HMs in roots was significantly higher (ANOVA, *p* = 0.0001, F ratio = 2911, df_bc_ (between columns) = 2, df_wc_ (within column) = 15, total df = 17) than in leaves and stems (Fig. 1a, b, e). Treatment of plants with mixed HM at a higher concentration of 50 μl, the plants did not survive. Comparing the control group with group-k (which was provided with a mixture of three HMs), group-k had highly significant absorption of HMs (ANOVA, *p* < 0.0001, F ratio 18296, df_bc_ = 5, df_wc_ = 30, total df = 35) of HMs.

Compared with the control group, Cd showed the highest percentage accumulation when used as a single HM at 25 or 50 μmol L^−1^ (groups-b, c) or as part of a mixture of HMs at 25 μmol L^−1^ (Table 1; groups-h, i, k). Concentration of Cd was greater in the roots than in the leaves (ANOVA, *p* < 0.0001, F ratio = 18296, df_bc_ = 5, df_wc_ = 30, total df = 35) when it was used as a single HM or when admixed with other HMs. However, in plants exposed to Pb in mixture or alone (groups-d, e, h, j, k; Table 1), both leaves and roots had a higher percentage accumulation, than plants exposed to Ni in mixture or alone (groups-f, g, i, j, k; Table 1). These results indicate that the order of HM accumulation in the plant was Cd > Pb > Ni.

**Table 1:** Percentage increase in heavy metal accumulation in *Conocarpus lancifolius* after exposure to single /mixed heavy metals at two different concentration

Group	Sample	Leaves			Stem			Roots		
		% increase			% increase			% increase		
		Cd	Ni	Pb	Cd	Ni	Pb	Cd	Ni	Pb
a	Control	0.77 ± 0.23	0.66 ± 0.12	0.85 ± 0.36	0.77 ± 0.23	0.66 ± 12	0.85 ± 0.36	0.77 ± 0.23	0.66 ± 0.12	0.85 ± 0.36
b, d, f	Cd, Pb, Ni-25	71.8 ± 2.1	48.1 ± 2.2	64.8 ± 2.4	75.4 ± 2.9	57.2 ± 1.9	66.3 ± 1.2	84.7 ± 2.4	52.2 ± 2.8	69.7 ± 2.5
c, e, g	Cd, Pb, Ni-50	76.5 ± 3.6	62.9 ± 3.1	72.4 ± 2.3	78.8 ± 2.7	71.3 ± 2.1	75.4 ± 2.2	86.9 ± 3.3	55.6 ± 2.3	76.4 ± 3.1
h	Cd-Pb-25	75.5 ± 2.9	-	74.0 ± 3.5	77.3 ± 1.8	-	77.3 ± 1.8	84.7 ± 2.7	-	76.4 ± 3.7
i	Cd-Ni-25	77.1 ± 3.4	56.4 ± 3.3	-	70.1 ± 2.1	60.1 ± 1.7	-	85.4 ± 3.1	56.0 ± 2.1	-
j	Pb-Ni-25	-	61.4 ± 2.7	77.4 ± 3.1	-	62.4 ± 2.3	80.1 ± 1.8	-	53.6 ± 2.5	77.3 ± 3.3
k	Cd-Pb-Ni-25	80.3 ± 4.2	54.8 ± 2.8	68.9 ± 2.9	84.3 ± 2.4	60.4 ± 2.7	75.4 ± 2.1	86.9 ± 3.7	55.6 ± 2.7	76.3 ± 3.8

Gr = Group, HM-25 and HM-50 (Gr: b, d, f; Gr: c, e, g, respectively) represent the single heavy metal treatment at concentrations 25 and 50 μM L^−1^, respectively. Mixed HMs (Gr: h-k) were used at 25 μM L^−1^. Data were collected on day 30 and compared with the control group (Gr: a). The data represent means ± SEM (*n* = 6)

Compared with the control plants, the accumulation of Pb in the treatment groups was significantly higher (ANOVA, *p* < 0.0001, F ratio = 2305, df_bc_ = 29, df_wc_ = 25, total df = 29) in roots than in leaves and stems of all treated plants (Fig. 1a, c, e). The BAFs and TFs suggested that Pb, as a single HM or admixed with other HMs, was better absorbed in the roots and transported to the leaves (Table 2, 3). Thus, an exposure of the plants to Pb alone at 25 or 50 μmol L^−1^ (groups-d, e, respectively, compared with the control group), caused a significantly higher Pb BAF (*t*-test, *p* = 0.0198, *t* = 3.77, df = 5, Table 2). The TF ratio of Pb (Table 3) was also significantly higher in group-d than in the control (*t*-test, *p* = 0.0001, *t* = 23). However, a comparison of the TFs for Cd (group-b, c) and Pb (group-d, e; Table 3), indicated that *C. lancifolius* is a better accumulator or transporter of Pb. The TF ratio for Pb was also significantly higher (*t*-test, *p* = 0.004, *t* = 5.1, df = 5) after the plants were exposed to mixed HMs (groups h–k compared with the control Table 3). This was also supported by a highly significant difference in HM accumulation between the control and groups-b, d, f and groups-c, e, g.

**Table 2:** Bioaccumulation factors (BAFs) and enrichment factors (EFs) for single and mixed heavy metals at two different concentrations and essential metal accumulation in leaves, stems, and roots of *Conocarpus lancifolius*

*Gp*	*Leaf*	*BAF*						*EF*					
		Cd	Pb	Ni	Se	Fe	Mg	Cd	Pb	Ni	Se	Fe	Mg
a	Control	0.77 ± 0.009	0.85 ± 0.008	0.66 ± 0.008	0.63 ± 0.011	0.91 ± 0.012	0.91 ± 0.015	0.77 ± 0.023	0.85 ± 0.022	0.66 ± 0.012	0.63 ± 0.22	0.91 ± 0.32	0.91 ± 0.22
b	Cd-25	1.05 ± 0.022	0.85 ± 0.007	0.69 ± 0.022	0.61 ± 0.007	0.97 ± 0.016	0.92 ± 0.011	2.41 ± 0.018	1.25 ± 0.008	1.84 ± 0.018	0.99 ± 0.33	1.45 ± 0.22	1.15 ± 0.22
c	Cd-50	1.21 ± 0.016	0.86 ± 0.211	0.71 ± 0.016	0.66 ± 0.004	0.98 ± 0.014	0.99 ± 0.019	2.52 ± 0.021	1.31 ± 0.031	1.73 ± 0.011	0.93 ± 0.29	1.51 ± 0.10	1.21 ± 0.19
d	Pb-25	0.75 ± 0.007	1.05 ± 0.212	0.69 ± 0.007	0.63 ± 0.012	0.96 ± 0.012	0.92 ± 0.006	1.13 ± 0.007	2.56 ± 0.020	1.95 ± 0.020	0.97 ± 0.15	1.33 ± 0.19	1.34 ± 0.11
e	Pb-50	0.76 ± 0.056	1.12 ± 0.114	0.72 ± 0.056	0.64 ± 0.006	0.92 ± 0.004	0.93 ± 0.007	1.22 ± 0.013	2.81 ± 0.019	1.58 ± 0.021	1.00 ± 0.11	1.47 ± 0.14	1.32 ± 0.16
f	Ni-25	0.71 ± 0.012	0.78 ± 0.092	1.05 ± 0.012	0.61 ± 0.031	0.92 ± 0.017	0.94 ± 0.015	1.15 ± 0.019	1.43 ± 0.013	2.21 ± 0.027	0.97 ± 0.16	1.11 ± 0.11	1.20 ± 0.13
g	Ni-50	0.72 ± 0.083	0.79 ± 0.076	1.12 ± 0.056	0.68 ± 0.063	0.99 ± 0.009	0.95 ± 0.012	1.22 ± 0.012	1.44 ± 0.011	2.95 ± 0.019	1.04 ± 0.10	1.61 ± 0.19	1.33 ± 0.18
h	Cd+Pb	1.22 ± 0.001	1.05 ± 0.091	0.77 ± 0.061	0.66 ± 0.021	0.98 ± 0.002	0.95 ± 0.021	2.21 ± 0.007	2.73 ± 0.019	1.84 ± 0.014	1.07 ± 0.11	1.60 ± 0.18	1.34 ± 0.11
i	Cd+Ni	1.21 ± 0.012	0.88 ± 0.073	1.11 ± 0.083	0.67 ± 0.065	0.96 ± 0.018	0.94 ± 0.019	2.23 ± 0.012	1.51 ± 0.020	2.80 ± 0.019	1.06 ± 0.09	1.53 ± 0.12	1.45 ± 0.17
j	Ni+Pb	0.76 ± 0.014	1.11 ± 0.096	1.09 ± 0.004	0.68 ± 0.062	0.95 ± 0.016	0.93 ± 0.020	1.54 ± 0.016	2.84 ± 0.009	2.90 ± 0.014	0.94 ± 0.06	1.45 ± 0.09	1.12 ± 0.19
k	Cd+Ni+Pb	1.25 ± 0.090	1.15 ± 0.047	1.09 ± 0.007	0.69 ± 0.011	0.95 ± 0.017	0.96 ± 0.016	2.22 ± 0.006	2.60 ± 0.012	2.63 ± 0.014	0.96 ± 0.05	1.50 ± 0.15	1.10 ± 0.23
Stems		BAF						EF					
a	Control	0.77 ± 0.007	0.85 ± 0.003	0.66 ± 0.008	0.63 ± 0.011	0.91 ± 0.012	0.91 ± 0.015	0.77 ± 0.043	0.85 ± 0.021	0.66 ± 0.014	0.63 ± 0.22	0.97 ± 0.22	1.11 ± 0.42
b	Cd-25	1.21 ± 0.011	0.86 ± 0.002	0.71 ± 0.006	0.62 ± 0.013	0.94 ± 0.015	0.95 ± 0.011	2.51 ± 0.025	1.22 ± 0.018	1.76 ± 0.028	1.07 ± 0.34	1.55 ± 0.12	1.18 ± 0.31
c	Cd-50	1.40 ± 0.015	0.80 ± 0.200	0.74 ± 0.001	0.66 ± 0.015	0.92 ± 0.011	1.10 ± 0.019	2.59 ± 0.032	1.33 ± 0.022	1.83 ± 0.022	0.98 ± 0.26	1.58 ± 0.14	1.28 ± 0.21
d	Pb-25	0.80 ± 0.012	1.30 ± 0.211	0.83 ± 0.003	0.64 ± 0.022	0.99 ± 0.012	0.95 ± 0.008	1.15 ± 0.012	2.66 ± 0.018	1.99 ± 0.031	0.96 ± 0.11	1.36 ± 0.20	1.44 ± 0.18
e	Pb-50	0.81 ± 0.061	1.42 ± 0.180	0.81 ± 0.021	0.66 ± 0.004	0.94 ± 0.005	0.98 ± 0.005	1.32 ± 0.018	2.91 ± 0.015	1.78 ± 0.025	1.02 ± 0.13	1.50 ± 0.14	1.35 ± 0.19
f	Ni-25	0.61 ± 0.014	0.61 ± 0.084	1.20 ± 0.023	0.63 ± 0.021	0.91 ± 0.016	0.94 ± 0.015	1.25 ± 0.016	1.52 ± 0.012	2.37 ± 0.024	0.98 ± 0.12	1.21 ± 0.21	1.25 ± 0.23
g	Ni-50	0.63 ± 0.063	0.64 ± 0.003	1.32 ± 0.080	0.70 ± 0.033	0.96 ± 0.008	0.99 ± 0.014	1.32 ± 0.014	1.71 ± 0.018	2.99 ± 0.017	1.01 ± 0.13	1.69 ± 0.21	1.36 ± 0.28
h	Cd+Pb	1.32 ± 0.011	1.20 ± 0.110	0.68 ± 0.052	0.68 ± 0.011	0.98 ± 0.002	1.01 ± 0.022	2.61 ± 0.012	2.85 ± 0.016	1.72 ± 0.011	1.04 ± 0.15	1.62 ± 0.24	1.35 ± 0.21
i	Cd+Ni	1.31 ± 0.016	0.84 ± 0.054	1.15 ± 0.041	0.69 ± 0.055	0.98 ± 0.018	1.12 ± 0.020	2.43 ± 0.023	1.57 ± 0.021	2.73 ± 0.016	1.01 ± 0.10	1.63 ± 0.18	1.55 ± 0.27
j	Ni+Pb	0.82 ± 0.017	1.28 ± 0.012	1.21 ± 0.012	0.71 ± 0.042	0.99 ± 0.016	1.11 ± 0.020	1.64 ± 0.027	2.91 ± 0.019	2.95 ± 0.014	0.98 ± 0.05	1.65 ± 0.14	1.22 ± 0.29
k	Cd+Ni+Pb	1.47 ± 0.056	1.37 ± 0.053	1.35 ± 0.034	0.72 ± 0.012	1.20 ± 0.015	1.23 ± 0.012	2.42 ± 0.028	2.98 ± 0.018	2.99 ± 0.019	0.99 ± 0.09	1.68 ± 0.18	1.23 ± 0.21
Roots		BAF							EF				
a	Control	0.77 ± 0.013	0.85 ± 0.016	0.66 ± 0.012	0.63 ± 0.010	0.91 ± 0.012	0.91 ± 0.022	0.77 ± 0.013	0.85 ± 0.016	0.66 ± 0.012	0.63 ± 0.022	0.91 ± 0.32	0.91 ± 0.22
b	Cd-25	2.33 ± 0.015	1.30 ± 0.019	1.07 ± 0.019	0.62 ± 0.030	1.09 ± 0.016	0.92 ± 0.018	3.03 ± 0.021	1.45 ± 0.019	1.67 ± 0.023	0.83 ± 0.018	1.15 ± 0.21	1.06 ± 0.18
c	Cd-50	2.63 ± 0.011	1.33 ± 0.024	1.46 ± 0.022	0.68 ± 0.019	1.30 ± 0.011	0.96 ± 0.019	3.42 ± 0.023	1.48 ± 0.021	2.29 ± 0.028	0.92 ± 0.015	1.38 ± 0.12	1.02 ± 0.21
d	Pb-25	0.88 ± 0.012	1.33 ± 0.020	1.32 ± 0.017	0.68 ± 0.012	1.17 ± 0.019	0.92 ± 0.021	1.15 ± 0.019	1.49 ± 0.023	2.07 ± 0.027	0.91 ± 0.019	1.24 ± 0.19	1.02 ± 0.20
e	Pb-50	1.16 ± 0.020	1.43 ± 0.019	1.30 ± 0.023	0.75 ± 0.010	1.26 ± 0.021	0.93 ± 0.019	1.50 ± 0.020	1.60 ± 0.017	2.04 ± 0.021	1.02 ± 0.012	1.33 ± 0.18	1.09 ± 0.15
f	Ni-25	1.15 ± 0.021	0.71 ± 0.012	2.01 ± 0.029	0.70 ± 0.013	1.22 ± 0.024	0.95 ± 0.016	1.50 ± 0.018	0.79 ± 0.014	3.16 ± 0.029	0.94 ± 0.016	1.30 ± 0.10	1.02 ± 0.18
g	Ni-50	0.97 ± 0.013	0.80 ± 0.017	1.65 ± 0.027	0.68 ± 0.011	1.27 ± 0.019	0.93 ± 0.012	1.26 ± 0.013	0.90 ± 0.019	2.59 ± 0.022	0.92 ± 0.013	1.35 ± 0.21	1.06 ± 0.19
h	Cd+Pb	2.35 ± 0.018	1.26 ± 0.017	1.45 ± 0.019	0.67 ± 0.018	1.38 ± 0.027	0.95 ± 0.019	3.06 ± 0.021	1.41 ± 0.017	2.28 ± 0.029	0.90 ± 0.019	1.46 ± 0.22	1.06 ± 0.24
i	Cd+Ni	2.29 ± 0.011	0.84 ± 0.011	1.94 ± 0.023	0.70 ± 0.010	1.19 ± 0.017	0.94 ± 0.021	0.99 ± 0.023	1.35 ± 0.021	2.51 ± 0.019	0.95 ± 0.016	1.41 ± 0.18	1.02 ± 0.11
j	Ni+Pb	0.76 ± 0.010	1.21 ± 0.021	1.60 ± 0.018	0.67 ± 0.019	1.33 ± 0.015	0.93 ± 0.020	2.98 ± 0.014	0.94 ± 0.027	3.05 ± 0.031	0.90 ± 0.018	1.26 ± 0.16	1.04 ± 0.14
k	Cd+Ni+Pb	2.27 ± 0.018	1.65 ± 0.025	1.94 ± 0.021	0.77 ± 0.016	1.52 ± 0.019	0.96 ± 0.015	2.95 ± 0.027	1.84 ± 0.025	3.05 ± 0.033	1.05 ± 0.021	1.62 ± 0.18	1.03 ± 0.18

Values represent means ± SEMs (*n* = 6) of the samples collected after 30 days of plant exposure. In column 2, the numbers in front of the metal show the concentration (μmol L^−1^) at which the plants were exposed. Mixed heavy metals were all at concentration 25 μmol L^−1^

**Table 3:** Translocation factors (TFs) for single/mixed heavy metal exposure in *Conocarpus lancifolius*

Group	Exposure	Cd	Pb	Ni	Se	Fe	Mg
a	Control	0.77 ± 0.23	0.85 ± 0.36	0.66 ± 0.12	0.63 ± 0.22	0.91 ± 0.32	0.91 ± 0.22
b	Cd-25	1.66 ± 0.21	0.65 ± 0.20	0.71 ± 0.19	0.68 ± 0.17	0.95 ± 0.22	0.91 ± 0.17
c	Cd-50	1.70 ± 0.27	0.66 ± 0.15	0.70 ± 0.21	0.71 ± 0.19	0.96 ± 0.21	1.02 ± 0.19
d	Pb-25	0.82 ± 0.19	1.61 ± 0.26	0.66 ± 0.17	0.63 ± 0.10	0.97 ± 0.28	0.99 ± 0.25
e	Pb-50	0.81 ± 0.17	1.89 ± 0.22	0.67 ± 0.16	0.62 ± 0.18	0.95 ± 0.19	1.03 ± 0.28
f	Ni-25	0.85 ± 0.19	0.66 ± 0.20	1.55 ± 0.29	1.20 ± 0.27	0.96 ± 0.21	1.05 ± 0.27
g	Ni-50	0.86 ± 0.21	0.69 ± 0.19	1.89 ± 0.23	1.43 ± 0.29	0.98 ± 0.16	0.96 ± 0.23
h	Cd+Pb	1.79 ± 0.17	1.88 ± 0.29	0.69 ± 0.19	0.72 ± 0.14	0.96 ± 0.17	0.92 ± 0.21
i	Cd+Ni	1.78 ± 0.28	0.69 ± 0.11	1.83 ± 0.12	1.53 ± 0.18	0.96 ± 0.16	0.99 ± 0.24
j	Ni+Pb	0.88 ± 0.18	1.89 ± 0.23	1.85 ± 0.18	1.64 ± 0.25	0.89 ± 0.21	0.96 ± 0.19
k	Cd+Ni+Pb	1.82 ± 0.27	1.85 ± 0.23	1.86 ± 0.21	1.61 ± 0.27	0.99 ± 0.19	1.04 ± 0.21

TF values represent means ± SEMs (*n* = 6) of the samples collected after 30 days of plant exposure. In column 2, the numbers in front of the metal show the concentration (μmol L^−1^) at which the plants were exposed. Mixed heavy metals were all at concentration 25 μmol L^−1^

The BAFs and EFs were used to assess the potential of plants to purge metals from the soil. In plants exposed to a mixture of two HMs containing Pb at 25 μmol L^−1^, (groups-h, j, leaf; Table 1), Pb facilitated the accumulation of Cd without affecting the accumulation of Ni. Relative to the levels in the control, when plants were exposed to mixed HMs containing Cd (groups-i–k; Table 1), the leaf BAF for Cd increased from 36.3% (in group-i) to 58.4%, and 62.3% in group-k, respectively. The presence of Pb also raised the leaf BAF for Ni (3.8%; group-f, j, k; Table 1). A similar synergistic cytotoxicity of Pb and Cd has been reported (Patra et al. 2011). Ni synergistic activity was seen in the BAF for roots (Table 1). The extraction factor (EF) ratios of plants exposed to single or mixed HMs were >1 (Table 1). In white-rot fungi, the potentiality of nickel and cadmium has been reported (Noormohamadi et al. 2018).

The TFs (0.77 in the control) were significantly higher (*t*-test, *p* = 0.0001, *t* = 2.48, df = 5) at 1.66 in plants exposed to Cd alone at 25 μmol L^−1^ (group-b, Table 3); similarly, TFs were significantly higher (*t*-test, *p* = 0.019, *t* = 3.37, df = 5) at 1.7 in plants exposed to Cd alone at 50 μmol L^−1^ (group-c). The TFs (Table 3) also showed that Cd significantly improved Ni translocation (group-i) after the plants were exposed to the 25 μmol L^−1^ mixture of the two HMs (*t*-test, *p* = 0.0007, *t* = 12.1, df = 5). Translocation of the metals, including essential metals (Fe, Mg, and metalloid Se), was also significantly higher (*t*-test, *p* < 0.02, df = 5) when plants were exposed to a mixture of the three metals (group-k). A higher TF (1.82) was obtained after the plants were exposed to a mixture of the three HMs at 25 μmol L^−1^. This indicated that the mixed metals had a synergistic effect on their translocation in *C. lancifolius*.

### Influence of HMs on soil pH and proline and protein content

**Fig. 2:**
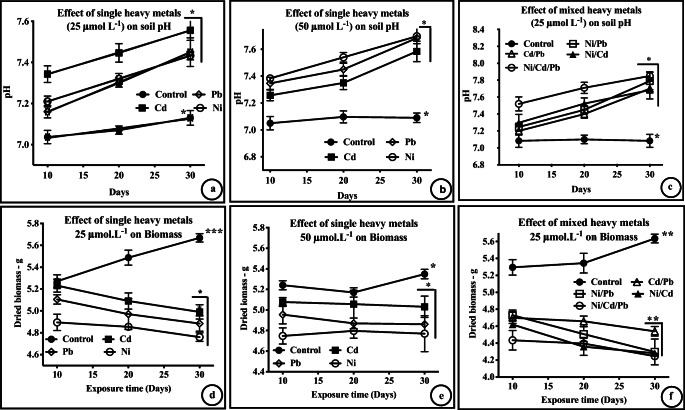
Heavy metal effect on the soil pH and biomass: Effect of heavy metals on soil pH **a**–**c** and biomass **d**–**f** of *Conocarpus lancifolius.* The changes in pH and biomass in response to single and mixed heavy metals were non-significant (t-test; *p > 0.05; *n* = 5). Data were compared with the respective control data (gp-a). *, insignificant differences (*p > 0.05)

**Fig. 3:**
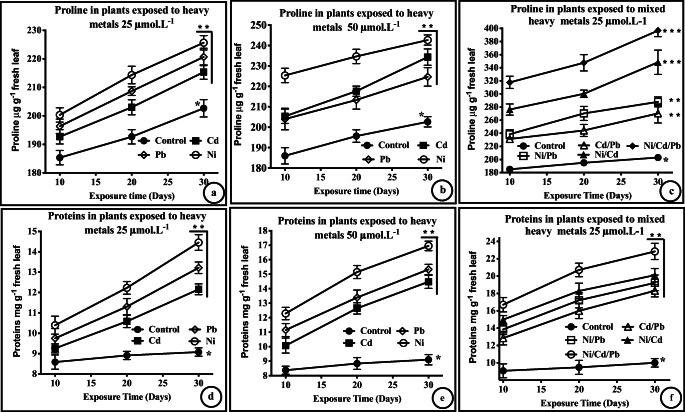
Accumulation of antioxidants in response to heavy metal stress. Accumulation of proteins **a**–**c** and proline **d**–**f** in plants exposed to single (25 or 50 μM L^−1^) and mixed (25 μM L^−1^) heavy metals. ** (*p* < 0.01) and *** (*p* < 0.001) represent significant and highly significant accumulations, respectively. Data were compared with the respective control data (group a) and a Student’s *t*-test (*n* = 6) was used to determine the p-values

After 30 d of the exposure to HMs, *C. lancifolius* showed a non-significant higher soil pH (*t*-test, *p* = 0.1317, *t* = 1.8, df = 5; Fig. 2a–c), and a non-significantly lower biomass (*t*-test, *p* = 0.2534, *t* = 1.29, df = 5; Fig. 2d–f); however, the levels of defense molecules, such as proteins and proline, were significantly higher than in the control (*t*-test, *p* = 0.2910, *t* = 1.18, df = 5; Fig. 3a–f).

## Discussion

Our results indicate that mixed HMs had synergistic toxicity even at lower concentrations, compared with single HMs at higher concentrations (Ucuncu et al. 2014). The toxicity of HMs results from the interaction between heavy metals and secondary metabolites, with the generation of higher OS, which has detrimental effects on the plant growth and development (Zhang et al. 2015; Wang et al. 2018; Zoufan et al. 2018). OS generation is induced by switching on the plant tolerance mechanisms, by producing the antioxidant constituents. Tomašević et al. (2004) reported that plant leaves may be used as good indicators of OS.

The accumulation of all three HMs was observed in the roots and leaves of *C. lancifolius*, indicating that the plant is a multi-HM tolerant shrub. This type of multi-HM accumulation is uncommon in plants and is mainly due to the synergistic toxicities of the HMs (Horvat et al. 2007). However, some plants are known to simultaneously accumulate Zn and Cu, but the increase in HMs in the vacuolar compartments and cell walls does not significantly damage the essential parts of the cell (Abdelkarim et al. 2019). Therefore, HM accumulation may take place in vacuoles where enzymes are least affected. Alternatively, elements may undergo oxidative modifications, such as the selenite–selenate oxidative transformation (Hawrylak-Nowak 2013) and Cr(VI)-Cr(II, IV); the former is highly toxic to plants while the latter is less toxic) Shanker et al. 2005).

An increase in the accumulation of HMs in *C. lancifolius* (Fig. 1, Table 1) implies that the plant is a hyperaccumulator of HMs, and that it can be used for the phytoremediation of contaminated soils. In hyperaccumulators, the accumulation of HMs in the roots is followed by the translocation of HMs into the leaves. Consistent with this, HMs accumulated more in the roots (Fig. 1a, b; Table 1) than in the leaves (Fig. 1c, d; Table 1). The higher accumulation of highly toxic Pb and Cd shows that *C. lancifolius* preferentially accumulated these HMs. This is because *C. lancifolius* has a high growth rate with a large green crown (Redha et al. 2019). Observations during this study revealed that *C. lancifolius* also has long hairy roots. The wide-spread hairy roots, a large crown, and fast growth are characteristic benchmarks of an HM hyperaccumulator.

Plant roots have selective uptake capabilities together with the translocation, bioaccumulation, and degradation abilities of the entire plant body (Rezvani and Zaefarian 2011). In phytostabilization process, roots bind the contaminants in the soil matrix, thus reducing their bioavailability. Plants also immobilize metal contaminants through absorption and accumulation causing precipitation in the root zone. This makes the accumulation of HM higher in roots. In addition, phytovolatilization through the overground parts of the plant is a common process lowering the concentration of HM in leaves and stems compared with roots (Tangahu et al. 2011).

Plants that have BAFs and TFs >1, metal concentrations of 1000–10,000 mg kg^−1^ in the aerial parts of the dried plant material, are classified as metal hyperaccumulators and five families of plants (Brassicaceae, Rubiaceae, Cunoniaceae, Salicaceae, and Euphorbiaceae) have been identified as hyperaccumulators of heavy metals (Van der Pas and Ingle 2019). *Thlaspi elegans*, a known hyperaccumulator, can accumulate 13,591–15,693 mg kg^−1^ of Ni, while the hyperaccumulator *Vaccinium myrtillus* L. is known to accumulate from 274 to 1,159 mg kg^−1^ of Mn (Kula et al. 2018). Thus far, ~400 plants have been classified as hyperaccumulators (Mahajan and Kuashal 2018). The BAFs for *C. lancifolius* were >1 (Table 2) for plants exposed to single or mixed HMs (i.e., Cd, Pb, and Ni, at 25 or 50 μmol L^−1^) indicating that *C. lancifolius* has a significant capacity to absorb and accumulate HMs, and can therefore be classified as a hyperaccumulator. The BAF, EF, and TF values were calculated as given above (line 182-186). The plants found in heavily polluted areas are known to have the values of BAF and TF >1 (Alhemaiti et al. 2018). In the present studies, exposures to HMs, the BAFs of Mn, a natural element found in soil, did not change, indicating its minimal toxicity to the photosynthetic apparatus of the plant. The BAFs for Fe, another natural element, which is absorbed from the rhizosphere as Fe^2+^, also remained unchanged, indicating that it does not affect the oxidative and photosynthetic status of the plant. Fe is an essential component of antioxidant enzymes such as catalase, ascorbate peroxidase, glutathione S-transferase (GST), superoxidase dismutase (SOD), and combats the OS generated by exposure to HMs; it also elevates the activities of these enzymes (Bielen et al. 2013). The BAFs for Se also remain unchanged. The BAFs for Fe, Mg, and Se were >1, which signifies efficient absorption of these metals in plant’s defense against the OS induced by the HMs. The BAFs for mixed HMs at 25 μmol L^−1^ were comparable with those of plants exposed to single HMs at 50 μmol L^−1^, indicating higher toxicity and OS induced by the mixed HMs.

The fact that the EF (Table 2) was the highest (>2) for the mixed HMs, suggests a synergistic effect of the HMs. The synergistic and antagonistic effects of multi-metal contaminated soil are due to an interaction between essential and non-essential metals, toxic and non-toxic metals polluting the soil (Ucuncu et al. 2014). The single HMs Cd, Pb, and Ni, and antioxidant metals Fe, Mg, and Se all had EFs >1. The antioxidant HMs further improved the plant defenses. The higher EFs indicate the greater absorption or accumulation of the HMs, distinguishing *C. lancifolius* as a hyperaccumulator of HMs. For the plants treated with mixed HMs, the higher EF (>2, Table 2) and TF (>1.8, Table 3) values, relative to the BAF (>1, Table 2), suggest their enhanced translocation and accumulation in the plant. The EFs and TFs were both >1 for mixed HMs, supporting the accumulation and translocation of the HMs in the aerial parts of the plant. The toxicity of the HMs is indicated by an amplified absorption of the antioxidant Se (EF 1.61, mixture of three mixed HMs). Huang et al. (2019) reported an increased uptake of Se in response to the OS induced by Cd. A nonsignificant increase (p > 0.05, *t*-test and ANOVA, compared with the control group) in the EF for Fe and unchanged EF for Mg also suggests that these metals have a defensive role against OS (Table 2). The elements involved in photosynthesis, respiration, and N assimilation (such as Fe and Mg) help to protect photosynthetic apparatus and improve plant growth by increasing tolerance to HMs. The fact that the BAF, EF, and TF values were >1 for plants treated with HMs alone and in mixtures indicates that *C. lancifolius* is an HM hyperaccumulator, and can thus be used for the phytoremediation of contaminated soils. An increased TF (>1, Table 3) indicates good translocation of HMs in hyperaccumulator plants (Kumar et al. 2018).

For plants exposed to Pb alone at 25 or 50 μmol L^−1^, the TF for Pb was significantly elevated (*p* < 0.02, *t*-test, groups-d, e compared with the control; Table 3), indicating that *C. lancifolius* is a good translocator of Pb. In mixtures of Pb with other HMs, the TF for Pb was significantly elevated (*p* < 0.01; *t*-test; groups-h, j, k; compared with the control; Table 3). Based on the TFs (Table 3), Pb translocation was significantly elevated (*t*-test, *p* < 0.03, df = 5) from 0.85 in the control to 1.61 in group-d and 1.89 in group-e, (that is, at both the low and high concentrations of Pb, 25 and 50 μmol L^−1^, respectively). The higher TF values in plants treated with mixed HMs suggest a synergistic effect of the HMs. Our results reveal a greater change in Pb translocation (groups-d, e; *p* = 0.01, n = 6, ANOVA) than in Ni or Cd translocation (Table 3). Pb mixed with other HMs also increased the translocation of Cd and Ni in plants (Table 3, groups-h, j, k). Thus, the exposure of the plants to Pb, mixed with Cd or Ni, (groups-h, j, k) at 25 μmol L^−1^, resulted in an accumulation of Pb that was comparable to the plants exposed to Pb alone at a higher concentration (50 μmol L^−1^, group-e). An exposure of the plants to the higher concentration of the single HM Cd (50 μmol L^−1^, group-c) resulted in a TF of 1.7, which was highly significantly elevated (*t*-test, p < 0.01, df = 5) relative to the control. Therefore, *C. lancifolius* is an excellent accumulator or translocator of Cd, Ni, and Pb (Table 2, 3).

The presence of one metal may affect the availability of another in the plant, indicating antagonistic or synergistic behaviors of metals (Raiesi and Sadeghi 2019). Thus, Pb and Ni were shown to have a synergistic effect on the translocation and accumulation of Cd. When Cd co-occurs with Ni, Cd absorption is stronger, and Cu has been shown to increase the toxicity of Zn in barley (Luo and Rimmer 1995). However, different oxidation states may make the metals less toxic by affecting their solubility and their bioavailability (Abedin et al. 2002).

Cross-tolerance of HMs is uncommon. For example, a species tolerant to Zn may be killed by Cu. However, some grasses and leguminous plants like *Lathyrus sativus* are known for their co-tolerance toward HMs (Liu et al. 2015; Abdelkarim et al. 2019). Nicholls and Mal (2003) reported that a combination of Pb and Cu kills the leaves and stems of *Lythrum salicaria*. In a study of six HMs in maize, the order of toxicity was established to be Cd > Co > Hg > Mn > Pb > Cr (Ghani 2010). We have previously reported the effect of HMs on the *C. lancifolius* photosynthetic apparatus (Redha et al. 2019).

Soil salinity is a persistent issue in Kuwait and the salinity varies with the geomorphological landscape (Redha et al. 2019; Bannari et al. 2020). Soil salinity increases the availability and toxicity of Cd and decreases the soil microbial respiration rate, microbial biomass, and enzyme activity (Raiesi and Sadeghi 2019). Metal availability from soil is known to depend on the soil properties, including solubility and soil surface area, and soil pH can significantly affect the accumulation of HMs in plants (Khatun et al. 2016; Alhemaiti et al. 2018). Soil pH is also an important factor for the growth and diversity of rhizospheric bacterial communities, and for the soil organic matter content, which are required for soil improvement and plant growth (Wang et al. 2019). Our results showed that soil pH was non-significantly elevated (*t*-test, *p* > 0.05, df = 5, compared with the control group) when *C. lancifolius* plants were exposed to single or mixed HMs (Fig. 2a–c).

Although a change in pH may have an insignificant effect on plant growth, its effects on rhizobacterial or fungal growth may be important; rhizobacteria and fungi play important roles in plant resistance to HM toxicity, by stimulating root exudates, thus affecting plant growth and biomass (Harter 1983; Šmejkalová et al. 2003; Friedlova 2010). For example, siderophores produced by soil microbiota improve the bioavailability of metals for plants (Huyer and Page 1988). Rhizodeposition of carbon may account for 5% of the total photosynthetically fixed carbon, but its composition and contents are sensitive to bacterial or fungal diversity. The concurrence of the rhizospheric microbiome and soil organic matter may control soil pH and transform or detoxify HMs (Berg and Smalla 2009). Siripan et al. (2018) demonstrated the use of rhizospheric bacteria for Cd toxicity alleviation in plants.

In our study, a nonsignificant elevation (*t*-test, *p* > 0.05, df = 5, control group compared with the treated group) was observed in the soil pH (Fig. 2a–c). Numerous factors may be involved in regulating soil pH. First, this regulation may be due to alterations in the soil physicochemical composition induced by the HMs, altering rhizospheric microbial composition and communities. Second, the regulation may change the oxidative state of the HM, making it more or less toxic. Thirdly, this regulation may be a result of the exudates from the plant roots in response to HM stress. Lastly, soil pH may induce tissue-specific transcriptional changes in ion transporters, affecting the Na^+^/K^+^ ratio (Elda et al. 2019).

Acidosis of soil can be triggered by the secretion of acidic components, such as shikimic, gallic, fumaric, acetic, oxalic, glutamic, and succinic acid, which significantly increases the soil microbial activity (Javed et al. 2017; Ray et al. 2017). However, there is an evolutionary divergence between species in terms of adaptive root exudation (Bowsher et al. 2016). Therefore, the control of soil pH is multifaceted, and not only controls the diversity of rhizospheric bacterial communities but also affects the availability of plant nutrients. The soil in Kuwait, in which *C. lancifolius* naturally grows and thrives, is moderately alkaline. In addition, Kuwait desert temperatures range from 10 to 50°C, and higher temperatures have been shown to help thermophilic bacteria and fungi thrive and produce antioxidant enzymes; an example of this is the SOD isolated from the thermophilic *Geobacillus stearothermophilus* (Gligic et al. 2000; Afzal et al. 2011). The soil in Kuwait is known to contain a diversity of thermophilic and moderately thermophilic bacteria, in the genera *Amycolatopsis*, *Chelativorans*, *Isoptericola*, *Nocardia*, *Aeribacillus*, *Aneurinibacillus*, *Brevibacillus*, *Geobacillus*, *Kocuria*, *Marinobacter*, and *Paenibacillus* (Al-Mailem et al. 2015). This microbial diversity plays an important role in the phytoremediation of soil contaminated with HMs.

The suitability of the experimental environment was also reflected by a nonsignificant decrease (*t*-test, *p* > 0.05, df = 5) in the biomass of *C. lancifolius* in response to single or mixed HM stress (Fig. 2d–f). Although Pb and Cd, at very low concentrations, are reported to be very toxic for plant growth (Ghani (2010), *C. lancifolius* seems to be tolerant and able to resist the toxicity of these single metals at high concentrations of 25 and 50 μmol L^−1^, corresponding to 7.70 and 15.42 mg L^−1^, respectively. In *C. lancifolius*, the long and highly branched root architecture augments the uptake of nutrients and other components from the soil. All of the above characteristics make *C. lancifolius* a remarkably suitable plant for the phytoremediation of crude-oil-polluted soil. Many fast-growing plants in the Brassicaceae family are good hyperaccumulators of HM (Ashraf et al. 2011), while plants, such as *Sedum alfredii*, are hyperaccumulators of more than one metal, e.g., Cd and Zn (Chibuike and Obiora 2014).

The toxicity of HMs in soil affects the morphology, biochemistry, biomass, growth, and development of plants (Zhang et al. 2011; Ghavri and Singh 2012; Gautam et al. 2016; Redha et al. 2019). In general, HMs reduce the biomass of plants and microbiomes by affecting the major metabolic processes, such as those involved in photosynthesis, production of growth hormones, uptake of micronutrients, and water interactions (Vernay et al. 2007; Rodriguez et al. 2012). In the present study, in exposure response to single HMs at 25 or 50 μmol L^−1^, a nonsignificant reduction (*t*-test, *p* > 0.05, df = 5) in plant biomass (Fig. 2d, e) was observed, comparable to that in plants exposed to mixed HMs at 25 μmol L^−1^ (Fig. 1f). This implies that minimal cell injury occurs in plants in response to single HM stress.

The above results prompted an investigation into other parameters that may be involved in the protection of *C. lancifolius* against HM stress. In plants, the amino acid proline regulates reactive oxygen species (ROS) and reactive nitrogen species (RNS) production, and accumulates under environmental stress, especially under drought and salt stress conditions (Székely et al. 2008; Liang et al. 2013; Mairiam et al. 2019; Hasanuzzman et al. 2020). Thus, the osmoprotective role of proline under abiotic and HM stress is well documented (Hossain et al. 2014).

In the present study, osmo-proline was found to significantly increase (t-test, *p* < 0.05, df = 5) in response to both concentrations (25 and 50 μmol L−1) following exposure to the single HMs (Fig. 3a, b). Proline accumulation was the highest in response to exposure to mixed HMs (groups-h–k, highly significant, *t*-test, *p* < 0.02, df = 5), which indicates augmented defenses against OS, caused by the mixed HMs (Fig. 3c). The HM-induced formation of free radicals is encountered by an increased interaction with proteins and carbohydrates for phyto-chelation, and altering anti-oxidant enzyme in plants (Chen et al. 2018; Kozal et al. 2018; Xu et al. 2016). In some plants, HMs such as Cd induce upregulation of certain genes that produce proteins which are used in antioxidant defense mechanisms and in the detoxification of the HMs (Chen et al. 2019).

The protein content of the plants exposed to HMs was significantly elevated (*p* < 0.05, *t*-test) in response to both concentrations of the single HMs (Fig. 3d, e, compared with the control group), and protein accumulation was significantly elevated (*p* < 0.03, n = 6, *t*-test) when plants were exposed to the mixed HMs (Fig. 3f). The accumulation of proteins may offer a defense against the OS caused by HMs, via chelation with the sulfhydryl groups of proteins, thus detoxifying the HMs (Mukhtiar et al. 2017). Specifically, Hsp70s is a multifunctional heat shock protein that is a central component of transmembrane protein transport; it also helps in protein folding in the native state, thus stabilizing multiple proteins (Mayer and Bukau 2005). In plants, Pb is known to up-regulate Hsp70s, and this upregulation is a marker for Pb-induced stress (Esposito et al. 2012). An upregulation of defense proteins and detoxification triggered by Cd is also known (Ahsan et al. 2007). Thus, our findings show that *C. lancifolius* is an effective hyperaccumulator of HMs, combatting HM-induced OS by accumulating proline, proteins, and antioxidant metals. These findings are important for the phytoremediation of HM-contaminated soils in Kuwait.

## Conclusion

The bioaccumulation, extraction, and transcription factors for heavy metals were all >1 indicating *C. lancifolius* is a hyperaccumulator when exposed to single or mixed HMs, and it can be used for the phytoremediation of crude oil contaminated soil and particulate matter that are hazardous to human health. Our results indicated that an exposure of the plants to mixed heavy metals was more detrimental than single heavy metal at the same concentration. The heavy metals uptake by *C. lancifolius* was not only concentration dependent but also depended if the plant was exposed to single or mixed heavy metals. In response to the oxidative stress imposed by the heavy metals, the plant defenses were upregulated by an elevation of antioxidants such as Fe, Mg, Se, osmoprotective proline, and proteins. The rhizospheric bacteria maintained the soil pH, and it was not conceited by the heavy metals resulting in an insignificant decrease in the plant biomass. Furthermore, the plant survived in the harsh desert environment and could thus be widely introduced for not only phytoremediation but also for greenery, shade, and to stabilize sand dunes. Further studies are warranted to examine the plant-metal interaction of other heavy metals present in crude oil.

## Data Availability

• The datasets used and/or analyzed during the current study are available from the corresponding author on reasonable request. • All data generated or analyzed during this study are included in this published article. • Data sharing is not applicable to this article as no datasets were generated or analyzed during the current study.
